# Schistosomiasis Mansoni-Recruited Eosinophils: An Overview in the Granuloma Context

**DOI:** 10.3390/microorganisms10102022

**Published:** 2022-10-13

**Authors:** Kássia K. Malta, Cinthia Palazzi, Vitor H. Neves, Yasmin Aguiar, Thiago P. Silva, Rossana C. N. Melo

**Affiliations:** Laboratory of Cellular Biology, Department of Biology, Federal University of Juiz de Fora (UFJF), Juiz de Fora 36036-900, MG, Brazil

**Keywords:** *Schistosoma mansoni*, schistosomiasis, eosinophils, granuloma, hepatic granuloma, histopathology, eosinophil degranulation, inflammation

## Abstract

Eosinophils are remarkably recruited during schistosomiasis mansoni, one of the most common parasitic diseases worldwide. These cells actively migrate and accumulate at sites of granulomatous inflammation termed granulomas, the main pathological feature of this disease. Eosinophils colonize granulomas as a robust cell population and establish complex interactions with other immune cells and with the granuloma microenvironment. Eosinophils are the most abundant cells in granulomas induced by *Schistosoma mansoni* infection, but their functions during this disease remain unclear and even controversial. Here, we explore the current information on eosinophils as components of *Schistosoma mansoni* granulomas in both humans and natural and experimental models and their potential significance as central cells triggered by this infection.

## 1. Introduction

Schistosomiasis, a neglected tropical disease caused by the trematode worms from the genus *Schistosoma*, persists as an illness of significant socioeconomic impact, with transmission being reported in 78 countries. Humans are infected after exposure to freshwater contaminated with free-swimming schistosome cercariae released by water snails (intermediate hosts). Human schistosomiasis is a complex disease that affects multiple organs—mainly, the liver and intestines—and has a wide range of clinical manifestations [1,2].

A key feature of the human infection with *Schistosoma mansoni* parasites, the only *Schistosoma* species that occurs in the Americas, is the development of marked eosinophilia. Following blood eosinophilia, eosinophils and other immune cells migrate to specific sites of infection, accumulating around parasite eggs that become trapped in host tissues [3,4,5]. These immune cell aggregates—termed granulomas—are compact, highly organized structures with a plethora of cells playing different roles while interacting with each other. Despite an extensive history of research undertaken to understand the role of eosinophils during *S. mansoni* infection, their functions remain unclear or even controversial, and there is considerable debate as to whether they act as effectors, immunomodulators, or merely remodeling operators [6,7,8,9]. Here, we explore the current information on eosinophils as components of schistosomal granulomatous inflammation and their potential significance as central cells triggered by *Schistosoma mansoni* infection.

## 2. Immunopathology of *Schistosoma mansoni* Infection

### 2.1. The Host Immune Response

*Schistosoma* worms have a life cycle involving an aquatic snail intermediate host and a definitive mammalian host. During contact with contaminated freshwater, schistosome cercariae actively penetrate the mammalian host’s skin, transforming into schistosomula forms of the parasite that migrate into the lungs through venous circulation and then to the liver, where the parasites mature. The adult *S. mansoni* worms mate by pairing and migrating to the mesenteric veins of the intestines, where oviposition occurs [1,2]. From there, eggs can be transported through the intestinal mucosa and expelled by the host [1,2,10].

The initial immune response of the acute *S. mansoni* infection is mostly caused by the schistosomula and the juvenile worm stages and is characterized by a typical T helper type 1 (Th1) profile with the expression of the pro-inflammatory cytokines tumor necrosis factor-alpha (TNF-α), interferon-gamma (IFN-γ), and the interleukins (IL) IL-1, IL-2, and IL-12 [10,11,12]. This Th1 reaction can cause dry cough, fever, and angioedema, accompanied by blood eosinophilia [1,13].

Soluble egg antigens (SEA) are strong inducers of type 2 immunity [14,15]. Thus, with the onset of the egg deposition, the immune response begins a progressive switch towards a T helper type 2 (Th2) profile, characterized by the presence of IL-4, IL-5, IL-10, and IL-13, together with the active production of immunoglobulin E [10,11,12,16]. Although the eggs are transported through the intestinal wall to be eliminated with the fecal material for the continuity of the *Schistosoma* life cycle, a large proportion of them can stay trapped in the intestinal wall or even be directed to other organs—mainly, the liver [17]. Eggs from *S. mansoni* can also be ectopically located in the spleen [18], lungs [18,19], reproductive system [20], and central nervous system [21,22].

With the disease progression, there is a downmodulation of the Th2 response and a minor increase in the Th1 environment, achieving a more balanced inflammatory profile [16]. This may be due to the action of T regulatory cells (Tregs) and B regulatory cells (Bregs), which reduce Th2 inflammation via an IL-10-mediated pathway [23,24,25,26], thereby immunoregulating the Th1/Th2 balance. This alteration in the immune profile marks the transition from the acute phase of schistosomiasis to the chronic condition [16]. In this phase, there is a reduction of the egg-induced inflammatory response, consequently changing the granulomatous inflammation, with granulomas tending to be smaller and more fibrous [27]. Indeed, our group has shown that, in experimental models of chronic hepatic schistosomiasis, there was a higher frequency of granulomas at the late stages of development compared to the acute phase [28]. 

### 2.2. The Granuloma Architecture 

Granulomas are the most prominent pathological feature of schistosomiasis that arises as a result of persistent antigenic stimulation [7,27,29]. *Schistosoma* granulomas can be defined as a compact assembly of inflammatory and resident cells forming a well-defined structure surrounding the parasite eggs [30,31]. T and B cells, as well as their subsets; neutrophils; eosinophils; basophils; mast cells; resident macrophages (Kupffer cells); inflammatory and differentiated macrophages (epithelioid and giant cells); hepatic stellate cells; and fibroblasts compose an intricate cell community within the *Schistosoma* granuloma [7]. 

The formation of granulomas during *Schistosoma mansoni* infection is considered a protective mechanism—a physical barrier between the egg and the surrounding tissue that minimizes tissue damage from products released by the parasite eggs [7,17,29,32,33]. In fact, the cell community stimulates and produces a collagen-rich extracellular matrix (ECM) within the granuloma, forming a tightly packed construct that isolates the *Schistosoma* egg, avoiding the contact of its antigens and excreted toxins with the host tissue [29]. Paradoxically, the extensive inflammation and collagen deposition caused by the granulomatous response are the main causes of tissue damage and pathology during schistosomiasis [29]. In the intestines, the granulomatous inflammation will lead to bleeding, microulcerations, and pseudopolyposis [1], while in the liver, it results in hepatomegaly and can generate periportal fibrosis with the occlusion of smaller portal branches, a severe pathology associated with portal hypertension and death [1,34,35].

The cell collection greatly varies among different *Schistosoma* species-induced granulomas. For instance, while, in *S. japonicum* granulomas, neutrophils are the most abundant cells, *S. mansoni* granulomas are characterized by massive eosinophil infiltrates [31]. 

*Schistosoma* granulomas are dynamic structures whose formation follows specific events associated with cell migration, cell–cell, cell–egg, and cell–ECM interactions [7,27]. Because of this dynamism and complexity, granulomas have a remarkable morphological variation, showing a variety of sizes and cellular compositions depending on their developmental stages [28,32,33]. 

**Figure 1:**
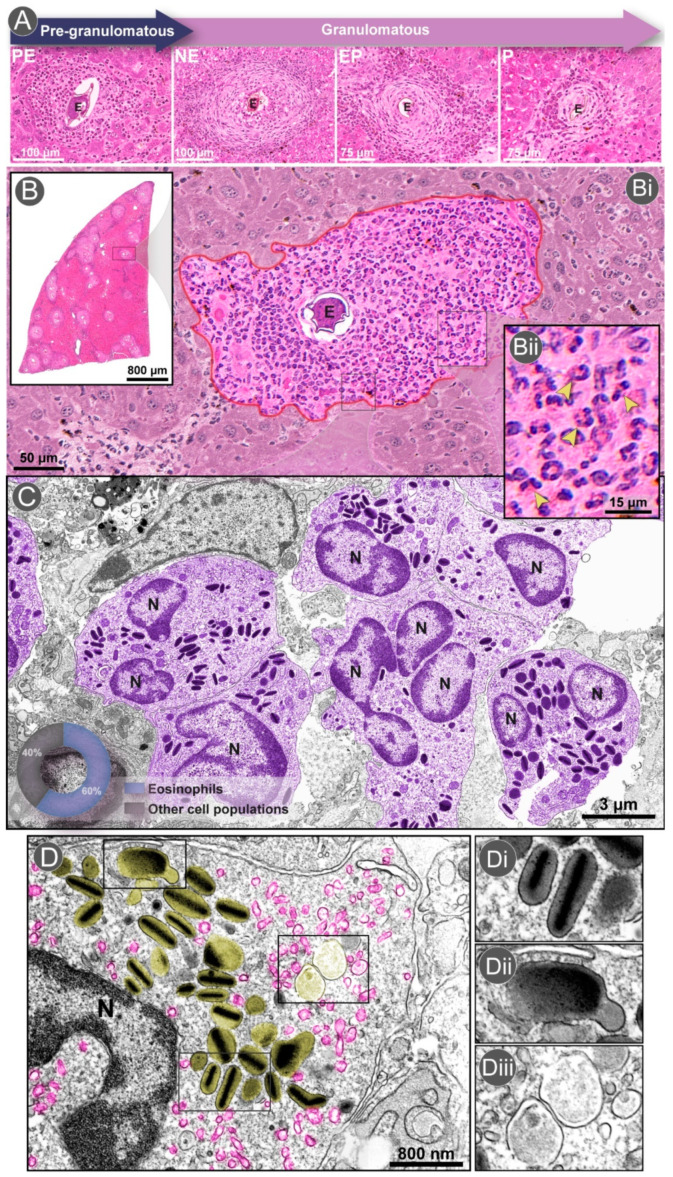
Eosinophils in a hepatic *Schistosoma mansoni* granuloma. (**A**) Evolutional stages of the hepatic granuloma and their features: Pre-granulomatous exudative (PE), with an initial infiltration of inflammatory cells in the organization process around the parasite egg (E); Necrotic-exudative (NE), with an area of necrosis in the periovular region around the egg (E) and numerous inflammatory cell types irregularly distributed on subsequent layers; Exudative-productive (EP), more organized and circumferential, with a rich structure of collagen and inflammatory cells concentrated in the periphery; and Productive (P), with a thick band of collagen fibers surrounding the egg (E) and a reduced number of inflammatory cells. (**B**) A representative whole-slide image of the hepatic tissue showing many granulomas (box). (**Bi**) A PE granuloma (outlined in red) forms around the parasite egg (E). (**Bii**) At a higher magnification, observe the accumulation of eosinophils (arrowheads). (**C**,**D**) Infiltrated eosinophils (colored in purple in **C**), seen under transmission electron microscopy (TEM), show their typical ultrastructure with a polylobed nucleus (N) and a high number of cytoplasmic-specific (secretory) granules (colored in yellow in **D**). Eosinophils represent 60% of all granuloma cells in hepatic NE granulomas [36]. (**D**) A representative eosinophil shows morphological signs of piecemeal degranulation (PMD), characterized by the presence of enlarged, non-fused granules releasing their content and a high number of transport vesicles (pink), predominantly around emptying granules (yellow). (**Di**) Intact granules with their unique morphology: a central well-defined electron-dense crystalline core and an outer electron-lucent matrix; (**Dii**,**Diii**) Swollen granules with disarranged cores and matrices denote PMD. Liver fragments from experimentally infected (acute phase) mice were prepared for light microscopy (**A**,**B**) and conventional TEM (**C**,**D**), as before [36]. Histological sections (**A**,**B**,**Bi**,**Bii**) were stained with Hematoxylin-Eosin. Panels (**D**–**Diii**) were republished from ref. [36] under the terms of the Creative Commons Attribution License 4.0 (CC-By).

Based on extensive histopathological studies, it is well documented that *Schistosoma* granulomas undergo different stages during their formation, progressing from a maturative to an involutional state (Figure 1A). Thus, two main stages are recognized in the granuloma construction: a pre-granulomatous stage, in which the inflammatory cells start their organization around the parasite egg, and a granulomatous stage, highly organized and characterized by progressive phases. Classically, the nomenclature for the *Schistosoma* granulomas, including the initial and three progressive stages, is the following: (i) pre- granulomatous exudative (PE), in which the first collection of cells accumulates in the host tissues around the parasite eggs; (ii) necrotic-exudative (NE), identified by a higher density and complexity of inflammatory cells that are irregularly distributed in different layers with a central necrotic area around the egg; (iii) exudative-productive (EP), a more organized granuloma with a well-defined circumferential aspect characterized by a rich structure of collagen fibers and inflammatory cells concentrated in the granuloma’s periphery; and (iv) productive (P), identified by the presence of small numbers of inflammatory cells and a thick band of collagen fibers surrounding the egg [28,32] (Figure 1A). 

Our group has provided the first detailed characterization of granulomas in the target organs (liver and intestines) of experimental models of schistosomiasis with the use of whole slide imaging (WSI) [28]. This technique allows for the scanning of the entire tissue sections for a comprehensive histopathological analysis, including the quantitative evaluation of granuloma morphological aspects (number, size, evolutional types, frequency, tissue areas occupied by them in the target organs, as well as the assessment of non-granulomatous inflammation). We showed that all four of these granuloma developmental stages are found within the liver, while only the PE and EP stages were clearly identified in the intestines [28]. This could be related to the more transient aspect of *Schistosoma mansoni*-induced intestinal granulomas.

## 3. Eosinophil Dynamics within the Granuloma

### 3.1. Eosinophil Recruitment and Accumulation

Eosinophils are actively recruited during schistosomiasis caused by the parasite *S. mansoni*, as extensively documented in humans [1,5,10,37,38,39] and experimental models [39,40,41,42,43,44]. Acute schistosomiasis is characterized by increased numbers of eosinophils in the circulation, peritoneal cavity, and target organs (mainly, the liver, intestines, and lungs), with blood eosinophilia being observed even before egg deposition in the target tissues [40,45,46,47,48]. Oviposition can further stimulate more pronounced blood eosinophilia, as noted in patients with acute infection [5,10,37]. Tissue eosinophilia is found during the early stages of granuloma formation within the inner perimeter of circumoval inflammation, as detected by immunolabeling with Siglec-F [49], an eosinophil surface receptor considered a marker for this cell [50,51]. The chronic phase is generally associated with reduced blood eosinophilia, since studies on the chronic phase of *S. mansoni* infection in humans have shown eosinophilia in less than 50% of subjects [52,53].

Histopathological quantitative analyses in experimental models showed that the eosinophil is the most abundant cell type in both the acute and chronic phases of the infection. In hepatic granulomas, eosinophils compose 60% of all cell populations in the acute phase (55 days post-infection) (Figure 1B,C) and 45% of all cells in the chronic phase (120 days post-infection) [28]. Prior studies have shown that, even in early infections (16 days post-infection), the proportion of eosinophils can reach 70% of granuloma cells [54]. Ultrastructural analyses performed by our group revealed that eosinophils form tight groups of cells within hepatic granulomas developed during acute schistosomiasis (Figure 1C) and are seen interacting with each other and with other inflammatory cells [36,55] (see Section 3.4). In human biopsies, eosinophils can also be observed in the proximity of eggs deposited in the liver and intestines [3,56,57]. The major basic protein (MBP) secreted by these cells was associated with the formation of the phenomenon termed Splendore-Hoeppli (asteroid bodies), which is characterized by an eosinophilic hyaline fringe, often with a radiating or starlike configuration, that surrounds schistosome eggs in granulomas [56]. However, the nature of the Splendore–Hoeppli reaction, which is also described in other infectious diseases around parasites, fungi, bacteria, or even inert materials, is not well understood [58]. 

Eosinophils are recruited into inflammatory sites in response to several chemoattractant molecules. In the initial phase of granuloma formation, CD4+ T lymphocytes and other inflammatory cells release chemokines such as CCL2, CCL3, CCL4, CCL5, CCL7, CCL11, CCL12, CCL17, CCL22, and CCL24, which bind to their cognate receptors at the eosinophil surface and regulate eosinophil migration [27,59,60,61,62,63,64]. CCL11 is particularly important for eosinophil migration from circulation [27]. Eosinophils are also chemically attracted by sensitization with antibodies and complement [40,45], as well as antigens released by parasite eggs trapped in the target tissue. However, SEAs alone are unable to chemoattract the high number of eosinophils observed in the infected tissues [45,65,66]. 

Signaling from T-lymphocytes and other immune cell populations, such as dendritic cells, is essential to attracting and accumulating eosinophils into the granuloma ecosystem. CD4+ T lymphocytes release IL-5, a key cytokine that attracts eosinophils to the inflammatory tissue sites and also contributes to increased blood eosinophilia in association with granulocyte-macrophage colony-stimulating factor (GM-CSF) under a Th2 environment [4,9,27,67,68,69]. Interestingly, the depletion of CD11c, a marker for dendritic cells, dramatically altered granuloma and hepatic cellularity, leading to decreased numbers of eosinophils and T cells [49,70]. 

### 3.2. Differential Distribution of Eosinophils within the Granuloma

In a recent review, we explored the hepatic *Schistosoma* granuloma as an “integrating and evolving ecosystem” with progressive structural and functional changes and not only as a place where a community of cells is settled [7]. In line with this perspective, it is not surprising that the numbers and distribution of eosinophils change during the granuloma’s development [33,71,72], i.e., these cells are not randomly distributed but rather occupy well-defined regions as the granuloma progresses. Thus, in the initial stage (pre-granulomatous) of the hepatic *Schistosoma* granuloma, eosinophils appear to be disorganized around the egg and in small numbers [28,71]. In the following PE stage, the numbers of eosinophils increase, and these cells are diffusely distributed throughout the granuloma, while in the NE, eosinophils are concentrated in the periphery and in the center of the granuloma. In the P stage, despite the beginning of fiber formation, eosinophils are still diffusely distributed throughout the granuloma [28,71]. Finally, it is described as a “healing phase” by fibrosis, in which they are concentrated in the periphery and in the center of the granuloma, being in smaller numbers than in the NE and P phases, but they are also seen inside the egg [71]. The meaning of this differential distribution within the granuloma awaits further investigation.

### 3.3. Are Eosinophils Produced Locally in Schistosoma mansoni Granulomas?

It is documented that extramedullary hematopoiesis can occur in association with *Schistosoma* granulomas, as observed in the livers of mice infected with *S. mansoni*—specifically, around blood vessels (perivascular hematopoiesis) and in the periphery of mature granulomas identified as EP [73,74,75]. These granuloma types are organized into three distinct zones separated by well-defined arrays of collagenous fibers: central or periovular, medial, and peripheral [28,71,74,75]. The peripheral zone is considered a perfect niche for the extramedullary hematopoiesis, since this region is populated with cells from myeloid lineage—mainly, eosinophils, neutrophils, and monocytes in different stages of maturation [75]. The clonal expansion of these cell lineages might be occurring within this granuloma zone in parallel to the release of immature cells from the bone marrow [75]. Hence, pluripotent precursors under the influence of hematopoietic growth factors might undergo local differentiation, thus potentially generating all the myeloid lineages [33,76,77]. Accordingly, by analyzing the ultrastructure of liver samples from experimental *S. mansoni* infection, we detected the presence of eosinophils exhibiting morphological features of immaturity (voluminous and less segmented nuclei, higher amounts of rough endoplasmic reticulum strands, and a small proportion of coreless secretory granules) as components of inflammatory infiltrates [78]. However, it is still unclear if these not completely differentiated tissue eosinophils denote cells undergoing extramedullary differentiation/maturation or merely cells that were released from the bone marrow with an unfinished process of maturation [78].

In mice deficient in CCL3, a chemokine involved in eosinophil maturation [63], both hepatic and pulmonary granulomas are smaller, and eosinophils show decreased peroxidase activity [79,80]. Moreover, other eosinophils, T cells, macrophages, hepatocytes, and Kupffer cells release in situ macrophage migration inhibitory factor (MIF), a molecule that participates in the IL-5-driven maturation of eosinophils and tissue eosinophilia associated with *S. mansoni* infection [81,82]. Matrix metalloproteinase 9 (MMP9), a protein produced by neutrophils and involved in modulation processes of hematopoietic function, is also produced within the granuloma [75]. Finally, another recent study shows that even liver cells from infected mice have a greater potential to produce IL-5 as early as week 4 of infection, which could support not only the rapid recruitment of eosinophils during granuloma initiation and development [49] but also the local eosinophil differentiation. Altogether, the granuloma microenvironment is considered a niche favorable for eosinophil differentiation and maturation, but future studies are needed for a better understanding of these events. 

### 3.4. Eosinophil Degranulation Mechanisms

A massive collection of proteins, including four cationic (basic) proteins, chemokines, growth factors, and many cytokines, are additionally stored as preformed products within the specific granules of eosinophils (reviewed in [64,78,83]). The high amounts of cationic proteins—specifically, MBP (also known as MBP-1 or PRG2), eosinophil cationic protein (ECP, also known as RNase3), EDN (also known as RNase 2), and eosinophil peroxidase (EPX, also known as EPO)—are responsible for the acidophilic nature of eosinophil secretory granules, which are easily identified in histological sections due to the high affinity for eosin (Figure 1(B–Bii)) and other acid stains. 

The role of eosinophils relies on their ability to secrete their granule-derived immune mediators and other proteins, which is collectively referred to as degranulation [78]. Eosinophils can secrete their granule contents through differential degranulation processes: (i) exocytosis, (ii) piecemeal degranulation (PMD), and (iii) cytolysis, all of them identified in detail only with the application of transmission electron microscopy (TEM) [78]. Exocytosis, which is characterized by granule–granule and granule–plasma membrane fusions, in general, is not a frequent mechanism found in vivo. On the other hand, PMD, which is characterized by the predominance of nonfused granules exhibiting content losses, and cytolysis, in which eosinophils release their granules after plasma membrane disruption, are much more described in vivo in a variety of eosinophil inflammatory responses/diseases [78,84,85,86,87]. PMD in humans is characterized by an increased formation of large, cytoplasmic vesiculotubular carriers termed eosinophil sombrero vesicles (EoSVs), which transport cytokines such as IL-4 and IFN-γ and cationic proteins such as MBP from granules to the extracellular medium [78,84,85,86,87].

In the context of the experimental hepatic *S. mansoni* infection in mice, quantitative analyses at the ultrastructural level identified PMD and cytolysis as the predominant secretory processes of eosinophils associated with this disease (Figure 1(D–Diii)) [36]. Classical features of PMD such as an amplified number of large vesicles (analogous to the human EoSVs) and the vesicular transport of MBP were also detected in eosinophils accumulated in the liver of infected animals [36]. Interestingly, in a recent work, we showed that not only the secretory granules but also the mitochondrial dynamics within inflammatory eosinophils respond to the acute *S. mansoni* infection in mice with increased cristae remodeling and inter-organelle contacts [51]. Our TEM analyses captured a significant increase in the numbers and volume of mitochondrial cristae in response to schistosomiasis [51]. Moreover, in this study, we identified an enhanced ability of mitochondria from activated eosinophils to interact with secretory granules, potentially influencing eosinophil immune responses during *S. mansoni* infection [51]. 

We have also investigated the intestinal biopsies of patients with chronic mild schistosomiasis, the common form of this disease found in endemic regions as a result of repeated exposures. PMD and, mainly, cytolysis were detected, while compound exocytosis was not observed as a relevant degranulation process occurring in vivo within human eosinophils recruited by chronic schistosomiasis [78]. 

### 3.5. Eosinophil Interaction with Other Cell Populations

**Figure 2:**
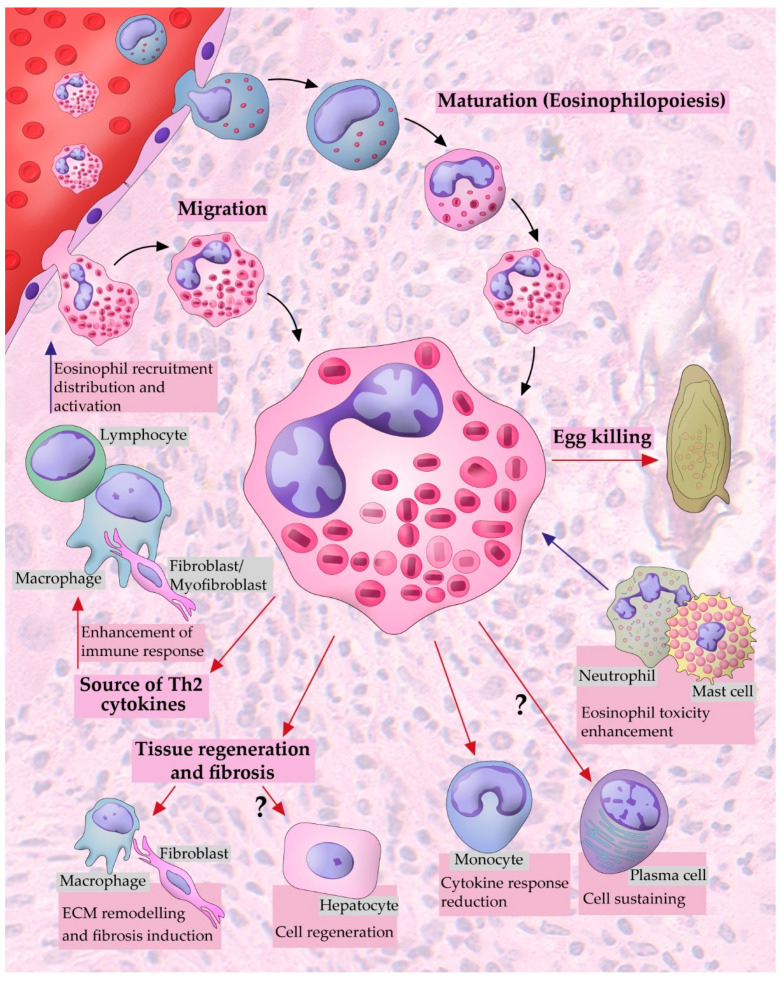
Eosinophil dynamics within a hepatic *Schistosoma mansoni* granuloma. Mature and immature eosinophils are recruited and migrate from the peripheral blood to target tissues, such as the liver. Undifferentiated eosinophils complete their maturation at the granuloma periphery (extramedullary hematopoietic sites). Eosinophils populate the granuloma and potentially interact with both the egg and other cell populations. Eosinophils may have the following possible functional roles and interactions within the granuloma: (i) destruction of the parasite egg through the secretion of cationic proteins [103]; (ii) interaction with neutrophils and mast cells with a resulting increase in the eosinophil toxicity against the parasite egg [94,95]; (iii) plasma cell sustaining; (iv) interaction with monocytes resulting in decreased monocyte responses [97]; (v) induction of hepatocyte proliferation/liver regeneration; (vi) interaction with macrophages and fibroblasts/myofibroblasts, with an impact on the extracellular matrix (ECM) remodeling and fibrosis induction [6,98]; (vii) enhancement of the immune response as a consequence of the eosinophil Th2 cytokines arsenal [90]; and (viii) interaction with lymphocytes, macrophages, and fibroblasts, which favors eosinophil recruitment, distribution, and activation [89,91].

A flow of information among different cells occurs within the boundaries of a *Schistosoma* granuloma, thus defining this structure as a real ecosystem with a diversity of cell populations interacting with each other and with the microenvironment [7,72,88]. Eosinophils constitute a vigorous cell population involved in the establishment and development of the granuloma, in which they form consistent cell–cell interactions [7] (Figure 2). However, considering the complexity of the *Schistosoma* granuloma as an interactive site, most eosinophil interactions are still poorly understood within this structure [7].

The release of IL-5 by CD4+ T lymphocytes, a key cytokine involved in the development and activation of eosinophils, leads to eosinophil recruitment to tissue sites in which granulomas are in the process of formation around parasite eggs [89] (Figure 1B). Once they reach the granuloma, this cell population is settled in an orderly and orchestrated way and is considered to contribute to the responsiveness against the parasite [33,47]. In parallel, eosinophils establish interactions with other cells, enhancing the level of the immune response with both the activation and immunomodulation of the granulomatous process [7,88]. Eosinophils are sources of Th2 cytokines such as IL-4, IL-5, IL-10, and IL-13 [90]. It is well known that these cells are capable of inducing an increase in the ratio of IL-13 versus IFN-γ, favoring an increase in the numbers of activated macrophages and fibroblasts, which are important cells in the process of tissue remodeling and fibrosis [6,91]. It is also documented that the interaction of eosinophils with macrophages contributes to the establishment and distribution of eosinophils inside the granuloma [91,92]. 

The interaction between eosinophils and mast cells is also reported within the granuloma [93]. In vitro studies suggest that such association helps eosinophils to potentiate the cytotoxic effect against the parasite since, in the absence of eosinophils or mast cells, a significant decrease in antiparasitic response effectiveness was observed [94]. In vitro experiments have also indicated a possible neutrophil–eosinophil interaction enhancing schistosomula killing [95]. The interaction with neutrophils increases the secretion of EPO by eosinophils, which potentiates the cytotoxic effect of eosinophil peroxidase [96].

Eosinophils seem to downmodulate monocytes [97]. In vitro observations showed that cytokines and chemokines produced by eosinophils could also be responsible for the reduced monocyte cytokine responses documented when monocytes were cultured with autologous eosinophils [97]. 

The interaction of eosinophils with myofibroblasts is likely involved in the immunoregulation of the inner workings of granulomas [98]. Studies in vitro showed that myofibroblasts release CCL11 and IL-5, crucial molecules for the recruitment and accumulation of eosinophils in granulomas. Thus, the interaction between myofibroblasts and eosinophils may promote eosinophil survival in *Schistosoma* granulomas [98]. Tissue eosinophilia is related to wound healing and repair in the intestine, liver, and lungs in several contexts [6]. Eosinophils produce enzymes that are critical for ECM remodeling and wound healing regulators such as resistin-like molecule-alpha (RELM-α), TGF-α, TGF-β, and fibroblast growth factors, which are associated with increased fibrosis [6,99,100].

Finally, eosinophils potentially interact with plasma cells and hepatocytes in the granuloma context. Eosinophils release some factors such as proliferation-inducing ligand (APRIL) and IL-6, which are responsible for maintaining long-lived plasma cells in the bone marrow [101]. In a situation of liver injury, similar to *Schistosoma*-induced tissue damage, eosinophils release IL-4, which binds to hepatocytes’ IL-4Rα receptors, thus promoting hepatocyte proliferation and consequent liver regeneration [6,102]. However, eosinophil–plasma cell or eosinophil–hepatocyte interactions have yet to be investigated in the *S. mansoni* granuloma. 

## 4. Eosinophils in Natural Models of Schistosomiasis

Although humans are the main definitive hosts for *S. mansoni*, some wild vertebrate animals, when in contact with *S. mansoni*-contaminated water, can eventually become naturally infected. For instance, primates, marsupials (skunks), ruminants, and rodents are considered permissive or reservoir hosts for *S. mansoni* [104,105,106,107]. These animal models have been used to study the basic biology, immunology, and pathogenesis of schistosomiasis and to explore how *S. mansoni* infects the hosts during the parasite cycle in natural environments. Moreover, because some murine models used in experimental infections have limitations in mimicking human disease [108], wild reservoirs are suggested as suitable biological models [109].

Rodents in Africa and neotropical areas can harbor *S. mansoni* infection [110,111,112]. In Brazil, the most important models of natural *S. mansoni* infection are the wild semiaquatic rodents, popularly known as water rats from the genera *Nectomys* and *Holochilus* [113,114], especially *H. sciureus* and *N. squamipes* [107,113,115]. Because of their semiaquatic habits, these rodents live in close contact with freshwater collections, an overriding factor for infection with the cercariae of *S. mansoni*. Furthermore, water rats complete the parasite cycle in the environment [115] and have a high tolerance to human presence. As a result, they play an important role in schistosomiasis transmission and epidemiology in endemic areas [107,116].

As noted, the granulomatous response triggered by the *S. mansoni* infection in both experimental models and in humans is characterized by a prominent accumulation of eosinophils around parasite eggs in the target organs (liver and intestines) [29,117]. Wild rodents naturally infected with *S. mansoni* follow the same pattern of eosinophil infiltration with the formation of typical granulomas, as seen in Figure 1A. Histopathological studies of target organs in naturally *Schistosoma*-infected *H. sciurus* and *N. squamipes* describe the initial stages of hepatic granulomas with marked inflammatory infiltrates rich in eosinophils [118,119,120]. An intense and diffuse infiltration of eosinophils surrounding eggs in the lamina propria of the *H. sciurus* esophagus has also been reported [121]. Costa Silva and colleagues [122] showed that the initial hepatic exudative *Schistosoma* granulomas in naturally infected *N. squamipes* are rich in diffusely distributed eosinophils, with a focal concentration around the eggs. In the same study, the late hepatic exudative granulomas, which had a predominance of large pigmented macrophages, showed a variable number of eosinophils, surrounded or not surrounded by mononuclear cells. This eosinophil distribution in the exudative granuloma was also found in the liver of *Calomys colossus*, another rodent model naturally infected with *S. mansoni* [123].

Previous studies from our group demonstrated that naturally infected *N. squamipes* can be a useful alternative model to help understand eosinophil functions in *S. mansoni* infection. By applying imaging analyses with WSI, we found a significantly lower infiltration of eosinophils in the target organs (liver and intestines) of *N. squamipes* compared to both acute and chronic experimental infection in mice. As noted, while eosinophils in the hepatic granuloma corresponded to 60% of all cells in the acute experimental infection in mice, eosinophils reached only 30% in the natural infection [28].

We showed a remarkably low intensity of the inflammatory response—both granulomatous and non-granulomatous inflammation—when the natural and experimental *S. mansoni* infections were compared. Indeed, it is widely recognized that water rats have a high tolerance to schistosomiasis, presenting a well-balanced relationship with the parasite [115]. These natural models have well-moderated *S. mansoni*-induced pathological features and life-long infections that do not affect their lifespan [124] or their reproductive capacity [125]. If lower eosinophil recruitment is influencing or limiting the severity of the disease in naturally *S. mansoni*-infected *N. squamipes*, it remains unclear.

## 5. Eosinophils in *Schistosoma mansoni* Infection: Effector or Immunomodulatory Cells?

Historically, eosinophils have been associated with the host’s response to helminth infections and with a host-protective and helminthotoxic function. Earlier in vitro studies have indicated an effector role of eosinophils against developmental stages of the *S. mansoni* parasite (schistosomula, pairs of adult worms, and eggs) [65,66,103,126,127,128]. These cells were considered “helminth killers”, capable of killing even the miracidia larvae inside the *S. mansoni* eggs through the secretion of cationic proteins—mainly, MBP [65,66,103,126,127,128]. However, the concept that eosinophils act as “defender effector cells” has not been supported by in vivo studies, and the eosinophil roles within the *Schistosoma* granuloma are still under debate (reviewed in [6,7,29,39,117,129]).

More recent studies have been changing the view of eosinophils as cytotoxic effector cells towards a more immunoregulatory role in both adaptive and innate immunity to parasite infections, including *S. mansoni* infection [97,130,131,132]. Swartz and colleagues [48] investigated the *S. mansoni* infection in two mouse models of eosinophil lineage ablation (ΔdblGATA and TgPHIL). In these models, no eosinophil-dependent differences in granuloma number, size, or fibrosis were observed, as well as no eosinophil-dependent differences in hepatocellular damage. Eosinophil ablation had no effect on worm burden and egg deposition and no impact on the traditional measures of the *S. mansoni* infection [48].

The authors also demonstrated a differential accumulation of mast cells within the granulomas of ΔdblGATA mice, which may compensate for the absence of eosinophils in granulomas [48]. Another study compared the liver immunopathological changes during experimental schistosomiasis in wild-type (WT) BALB/c mice and BALB/c mice selectively deficient in the differentiation of eosinophils (ΔdblGATA) [132]. While eosinophil differentiation had no effect on parasite egg retention in the liver, the authors reported a significant change in the liver immune response and tissue damage, resulting in significantly lower liver concentrations of IL-5, IL-13, IL-33, IL-17, IL-10, and TGF-α and higher concentrations of IFN-γ and TNF-α when compared to (WT) mice [132]. Moreover, the absence of eosinophils resulted in a higher mortality rate in mice infected with a high parasite load. Therefore, these data indicate that eosinophils participate in the establishment and/or amplification of liver Th2 and regulatory responses induced by *S. mansoni*, which is necessary for the balance between liver damage and fibrosis, which in turn is essential for modulating disease severity [132]. As a source of Th2 cytokines, eosinophils have also been shown to play a role in tissue remodeling and repair in murine models of infection by directly driving IL-4-mediated wound repair and regeneration as a post-toxin injury response in the hepatic tissue [102,132].

## 6. Conclusions

In summary, the dense population of eosinophils triggered by *S. mansoni* infection has been associated with multiple functional roles, including granuloma formation and the protection of the parenchymal tissue [29], the destruction of the entrapped eggs [103], the excretion of the eggs [17,88], remodeling and repair [6,102,133], and immunomodulation [132]. However, there is still a lack of clarity regarding the primary role of eosinophils in the *S. mansoni* infection, likely because their roles have been addressed as an individual population, which does not reflect the whole system (granuloma) in which eosinophils are inserted [7]. Lenzi and collaborators have demonstrated that more than 40,000 cells can populate *Schistosoma* granulomas such as the hepatic granuloma and that internal conditions regulate a robust network of cell–cell and cell–ECM interactions [32,33,72]. As *Schistosoma* granulomas are far more complex than merely structural compartments, studying them as ecological ecosystems with multi-directional interactions would provide a better understanding of eosinophil functions in this context [7]. Furthermore, future studies of eosinophil subpopulations would shed more light on the enigmatic role of these cells in *Schistosoma* granulomas, which is consistent with current concepts of the existence of phenotypically distinct eosinophils in tissues [55,78,134].

## Abbreviations:

ΔdblGATA	Lineage-ablated delta bone marrow progenitors
BALB/C	Albin Laboratory-Mouse
Bregs	Lymphocyte B Regulatory
CCL2	C-C Motif Chemokine Ligand 2
CCL3	C-C Motif Chemokine Ligand 3
CCL4	C-C Motif Chemokine Ligand 4
CCL5	C-C Motif Chemokine Ligand 5
CCL7	C-C Motif Chemokine Ligand 7
CCL11	C-C Motif Chemokine Ligand 11
CCL17	C-C Motif Chemokine Ligand 17
CCL22	C-C Motif Chemokine Ligand 22
CCL24	C-C Motif Chemokine Ligand 24
CCL26	C-C Motif Chemokine Ligand 26
CD11c	Marker for dendritic cells
CD4+ T	Lymphocyte T Cluster of Differentiation 4
ECM	Extracellular Matrix
ECP	Eosinophil Cationic Protein
EDN	Eosinophil-Derived Neurotoxin
EP	Exudative-Productive stage of granuloma development
EPO	Eosinophil Peroxidase
GM-CSF	Granulocyte-macrophage colony-stimulating factor
IL	Interleukin
IL-1	Interleukin Type 1
IL-2	Interleukin Type 2
IL-4	Interleukin Type 4
IL-5	Interleukin Type 5
IL-6	Interleukin Type 6
IL-10	Interleukin Type 10
IL-12	Interleukin Type 12
IL-13	Interleukin Type 13
IL-17	Interleukin Type 17
IL-33	Interleukin Type 33
IL-4Rα	Interleukin Type 4 Receptor-Alpha
IL-5Rα	Interleukin 5 Receptor Alpha
IFN-γ	Interferon Gamma
MBP	Major Basic Protein
MIF	Migration inhibitory factor
MMP9	Matrix Metallopeptidase 9
NE	Necrotic-Exudative stage of granuloma development
P	Productive stage of granuloma development
PE	Pre-Granulomatous Exudative stage of granuloma development
PMD	Piecemeal Degranulation
RELM-α	Resistin-Like Molecule-alpha
SEA	Soluble Egg Antigens
TEM	Transmission Electron Microscopy
TGF-α	Transforming Growth Factor alpha
TGF-β	Transforming Growth Factor beta
TgPHIL	Transgenic mice that lack eosinophils
Th1	Lymphocyte T Helper Type 1
Th2	Lymphocyte T Helper Type 2
TNF-α	Tumor Necrosis Factor Alpha
Tregs	Lymphocyte T Regulatory
WSI	Whole Slide Image
WT	Wild-Type
